# *Bombyx mori* miR-2845 represses the expression of fibroin light chain gene both *in vitro* and *in vivo*

**DOI:** 10.1371/journal.pone.0261391

**Published:** 2021-12-16

**Authors:** JingYi Huang, YanHua Chen, Juan Zhu, MeiXian Wang, ShunMing Tang, YongZhu Yi, XingJia Shen

**Affiliations:** 1 Jiangsu Key Laboratory of Sericultural Biology and Biotechnology, College of Biotechnology, Jiangsu University of Science and Technology, Zhenjiang, China; 2 Key Laboratory of Silkworm and Mulberry Genetic Improvement, Ministry of Agriculture and Rural Affairs, Sericultural Research Institute, Chinese Academy of Agricultural Sciences, Zhenjiang, China; East China Normal University School of Life Sciences, CHINA

## Abstract

To study the regulatory function of *Bombyx mori* (*B*. *mori*) miRNAs (bmo-miR) on the expression of fibroin light chain gene (*BmFib-L*), the 3’UTR of *BmFib-L* mRNA was used as the target for online prediction of miRNAs from miRBase using RNAhybrid Software, and miR-2845 was screened out. First, the expression profiles of miR-2845 and *BmFib-L* in larvae of the 5th instar were analyzed by Real-time quantitative PCR (RT-qPCR). Then recombinant plasmids (pcDNA3.0-pre-miR-2845 and pGL3.0-*BmFib-L*) were constructed to use for the expression of miR-2845 and *BmFib-L* 3’UTR, respectively. Cellular-level functional verification of miR-2845 on *BmFib-L* was carried out using multiple experimental methods (including dual luciferase reporter vectors, artificially synthesized mimics and inhibitors, and target site mutations). Finally, *in vivo* functional verification was performed by injecting the recombinant vector in 5th instar larvae. *BmFib-L* expression levels were detected using RT-qPCR in the posterior silk glands (PSG) of the injected larvae. Results showed that the expression of miR-2845 increased between the 1^st^ and 5^th^ day in 5^th^ instar larvae, but began to decline on the 5^th^ day, while the expression of the target gene *BmFib-L* increased sharply. This suggests that miR-2845 and *BmFib-L* expression levels show opposing trends, implying a negative regulatory relationship. In BmN cells, miR-2845 significantly down-regulated the expression of *BmFib-L*; the inhibitory effect of miR-2845 on *BmFib-L* was disappeared after mutation of the targeting site on 3’UTR of *BmFib-L*; in individuals, miR-2845 significantly down-regulated *BmFib-L* expression levels. Our results provide new experimental data for clarifying the molecular regulation mechanism of silk protein expression.

## 1. Introduction

MicroRNAs (miRNAs) are a class of endogenous non-coding small RNAs that have lengths between 19–22 nts. They are produced from a 70–80 nts precursor (pre-miRNA), and have a hairpin loop structure [1]. The 5’ end phosphate group and the methylated 3’end hydroxyl group are unique signs of mature miRNAs, which make them distinguished from most of the oligonucleotides [2]. They are widespread in multicellular organisms [3], and most of them exist in the form of gene clusters in the genome [4]. MiRNAs are highly conserved during biological evolution [5], and play important regulatory roles by complementary binding with mRNAs to inhibit the expression of target genes at the post-transcriptional level [6, 7]. They are involved in a wide variety of functions, including cancer cell proliferation, inhibition of cell senescence and other physiological processes [8, 9].

*B*. *mori* is an economically important insect, and a common model organism. Its silk glands have a strong ability to synthesize silk proteins. Silk fibroin is the main component of silk protein and is synthesized in the posterior silk glands (PSGs). It is composed of a heavy chain (BmFib-H), a light chain (BmFib-L) and a BmP25 [10]. Studies show that the three silk fibroin genes have similar regulatory mechanisms [11]. The protein factor BMFA not only can bind to *BmFib-L*, but can also down-regulate *BmP25* [12]. Silk gland-specific factor (SGFB) is a silk gland-specific factor, which is the main regulatory factor of *P25* gene in the posterior silk glands [13]. SGF-1 is one of the SGFB factors, and is involved in the regulation of the *Bombyx sericin-1* and *BmFib-H* genes [14, 15]. Silk protein genes are expressed in all stages of larval development, but have lower expression levels in the molting and metamorphic stages [16]. A large number of miRNAs have been identified throughout *B*. *mori* different developmental stages [17, 18], indicating that bmo-miRs play important roles in the regulation of growth and development. Among these, a few bmo-miRs related to the regulation of silk protein gene expression have been reported. Bmo-miR-0001 and bmo-miR-0015 down-regulate *BmFib-L* expression [19], and bmo-miR-275 represses Bombyx mori sericin gene 2 (*BmSer-2*) expression [20]. bmo-miR-2739 also up-regulates *BmFib-H* expression [21]. However, the intricacies of the silk protein expression regulatory network and its regulatory mechanism have not yet been fully elucidated.

In order to explore the underlying mechanism of miRNA regulation of silk protein expression, in this bioinformatics analysis study, the seed sequence of bmo-miR-2845 selected from the miRBase database (http://www.mirbase.org/) completedly matched the *BmFib-L* 3’UTR. We constructed miR-2845 expression vector using pcDNA3.0-pre-miR-2845 and *BmFib-L* 3’UTR recombinant plasmid pGL3.0-*BmFib-L*. At the cellular level, the regulatory function of miR-2845 on *BmFib-L* was verified through the dual fluorescence reporter system and overexpression and inhibition of expression using synthesized mimic and miR-2845 inhibitors. We also mutated the binding site for further verification. Finally, we verified the function at the individual level using the injection method. These results indicate that miR-2845 suppresses the expression of *BmFib-L* both *in vitro* and *in vivo*.

## 2. Materials and methods

### 2.1 Materials, reagents and instruments

The P50 silkworm strain was cultivated by Sericulture Research Institute, Chinese Academy of Agricultural Sciences. The larvae were fed with fresh mulberry leaves indoors at 25°C and 80% relative humidity.

*B*. *mori* ovary-derived BmN cells, plasmid pRL-CMV containing a Renilla luciferase gene, and expression vector pcDNA3.0[*ie*1-*egfp*-SV40] and pGL3.0[*A*3-*luc*-SV40] were constructed and preserved in our laboratory [21]. These vectors contain EGFP reporter genes and luciferase reporter genes, respectively. Primer synthesis and DNA sequencing were completed by Zhejiang SunYa Biotechnology. Total RNA extraction kit and reverse transcription kit were purchased from TaKaRa (Dalian, China). Plastic recovery and Plasmid Extraction Kit were purchased from Sangon Biotech (Shanghai, China). miR-2845 mimic and inhibitor were synthesized by RiboBio (Shanghai, China). A dual Luciferase Assay kit (Dual Luciferase® Reporter Assay System) was purchased from Promega (Beijing, China). Transfection reagent Entranster^TM^‐H4000 was purchased from Engreen (Beijing, China). UltraSYB Mixture and miRNA cDNA Synthesis Kit were products of Beijing ComWin Biotech.

The T100^TM^ gradient PCR instrument was manufactured by Bio-Rad Laboratories (Shanghai, China). The He-120* Multi-functional horizontal electrophoresis tank that we used was produced by Tanon Science & Technology (Shanghai, China). The 20/20 Luminometer luciferase detector was manufactured by Promega. The LightCycler® 96 quantitative fluorescence PCR system was produced by Roche (Basel, Switzerland).

### 2.2 Bioinformatic prediction of candidate miRNA

The *BmFib-L* 3’UTR sequence (accession number NM_001044023) was obtained from the NCBI (https://www.ncbi.nlm.nih.gov/) and all sequences of *B*. *mori* mature miRNAs were obtained from the miRBase (http://www.mirbase.org/). Based on seed sequence pairing levels from the 2nd to 8th bases of the 5’ end, a folding free energy < −20.0 kcal/mol(Rehmsmeier et al., 2004), a candidate miRNA, bmo-miR-2845, which may have regulatory effects on *BmFib‐L*, was screened out using RNAhybrid Software (https://bibiserv.cebitec.uni-bielefeld.de/rnahybrid/).

### 2.3 Extraction of RNAs from larval tissues

Total RNAs were extracted from the head, epidermis, malpighian tubule, midgut, trachea, hemocyte, fat body, testis, ovary, anterior silk glands (ASG), middle silk glands (MSG), and posterior silk glands (PSGs) of 5th instar day-3 larvae, and PSGs in 5th instar from day-1 to day-7. The total RNAs of corresponding tissues were extracted respectively according to the methods described in the RNAiso (TaKaRa) kit, and stored at -80°C.

### 2.4 Identification and analysis of expression profile of bmo-miR-2845 and *BmFib-L*

Specific primers for bmo-miR-2845 were designed. The upstream primer was originated form the mature miRNA by removing 6 bases at the 3’ end, and the downstream primer was the universal primer [22] (Table 1). PCR-derived DNA fragments (60–80 bp in length) were recovered after electrophoresis based on DNA Markers, and were cloned into pMD-19T vectors and sequenced. The total RNA extracted in step 2.3 was reverse transcribed with a reverse transcription kit in order to analyze the expression characteristics of bmo-miR-2845 and *BmFib-L*. *BmA3* served as an internal reference gene.

**Table 1:** Primer sequences.

Gene	Primer	Primer sequences (5’-3’)
*BmA*3	Forward	GGATGTCCACGTCGCACTT
	Reverse	GCGCGGCTACTCGTTCACT
Bmo-miR-2845	Forward	cagcCGTTGCCAGCTGCTG
	Reverse	GTGCAGGGTCCGAGGT
Pre-miR-2845	Forward	cccaagcttTCGACCAATGCTCCACCAAG
	Reverse	cgcggatccGGCCCTACGGTTTCTTTCGT
*BmFib-L* 3’UTR	Forward	tctagaATAAGAACTGTAAATAATGTA
	Reverse	ggccggccATCTGGAAAACTGGATACA
*BmFib-L*	Forward	TGCAAATTGTGTTTGCGTTAGG
	Reverse	CCAAAATGAAAAGCCGTGTCG

Note: lower case letters indicate protective bases or restriction sites.

### 2.5 Construction of recombinant expression vectors of pre-miR-2845 and *BmFib-L* 3’UTR

The pre-bmo-miR-2845 sequence was obtained from the NCBI and had about100 bp at both flanks. Primers were designed using NCBI Primer BLAST (Table 1). The pre-miR-2845 PCR product was cloned into pcDNA3.0-egfp vector after double digestion of *BamH* I and *Hind* Ⅲ to construct pcDNA3.0 -pre-miR-2845. Similarly, the *BmFib-L* 3’UTR recombinant expression vector pGL3.0-*BmFib-L* was constructed using restriction endonuclease *Xba* Ⅰ and *Fse* Ⅰ. Then recombinant expression vectors were identified by double digestion and sequencing. Their expression activities were validated in BmN cells by transfecting the vectors and tested by Real-time quantitative PCR (RT-qPCR) and luciferase assay, respectively.

### 2.6 Verification of regulation of bmo-miR-2845 on *BmFib-L* in BmN cells

To validate the regulatory capabilities of bmo-miR-2845 on *BmFib-L* in BmN cells, we paved well-grown BmN cells into a 12-well plate one day in advance to make them adhere to the walls and reach a density of about 80%. BmN cells were co-transfected with a total of 1.6μg plasmid DNA mixture of pcDNA3.0-pre-miR-2845 + pGL3.0-BmFib-L+ pRL-CMV at a ratio of 4:4:2, according to the transfection kit instructions (treatment group). Plasmid pRL -CMV was used as an internal reference. Cells co-transfected with mixture of pcDNA3.0-egfp + pGL3.0-BmFib-L+ pRL-CMV served as the control group. At 48 h post-transfection, observe the expression rate of *egfp* under a fluorescent microscope in order to evaluate transfection efficiency. Cells were collected and detected for luciferase activity using a Dual-Luciferase Reporter Assay System to explore the regulatory effect of miR-2845 on *BmFib-L*. Ratios of firefly luciferase activity to Renilla luciferase activity represented relative activity.

To further validate the regulatory function of miR-2845 on *BmFib-L*, both mimic (chemically synthesized double strand of mature miR-2845) and inhibitor (chemically modified complementary single stranded of mature miR-2845) were synthesized and diluted to 100nmol/L and 200nmol/L, respectively. We set up four groups of experiments: 10μL mimic + 0.64μg pGL3.0-BmFib-L+ 0.32μg pRL-CMV, 10μL inhibitor + 0.64μg pcDNA3.0-pre-miR-2845 + 0.64μg pGL3.0-BmFib-L+ 0.32μg pRL-CMV, 0.64μg pcDNA3.0-egfp + 0.64μg pGL3.0-BmFib-L + 0.32μg pRL-CMV and 0.64μg pcDNA3.0-pre-miR-2845 + 0.64μg pGL3.0-BmFib-L+ 0.32μg pRL-CMV. After 48 h of transfection, cells were collected and detected for luciferase activity using the Dual-Luciferase Reporter Assay System on a 20/20 Luminometer. The relative ratios of firefly luciferase activity to Renilla luciferase activity were used to validate the regulatory functions of miR-2845, and of the mimic and inhibitor on *BmFib-L*.

### 2.7 Mutation of bmo-miR-2845 targeting site on *BmFib-L* 3’UTR

To verify the accuracy of the bioinformatic prediction, the predicted targeting site on *BmFib-L* 3’UTR was mutated by using a mutant sequence (Sangon Biotech). Using the mutant sequence, we constructed a mutant recombinant expression vector pGL3.0-mut-BmFib-L. BmN cells were co-transfected as method above to verify the regulatory effects of miR-2845 on the mutated *BmFib-L* 3’UTR with following differing transfection solutions: pcDNA3.0-egfp + pGL3.0-mut-BmFib-L + pRL-CMV; pcDNA3.0-pre-miR-2845 + pGL3.0-mut-BmFib-L + pRL-CMV; mimic + pGL3.0-mut-BmFib-L + pRL-CMV and inhibitor + pcDNA3.0-pre-miR-2845 + pGL3.0-mut-BmFib-L + pRL-CMV.

### 2.8 Regulation of bmo-miR-2845 on *BmFib-L* in larvae

Since BmN cells are derived from ovaries, which cannot completely simulate the situation *in vivo*, the regulatory functions of bmo-miR-2845 on *BmFib-L* were further verified at the individual level. Day-2 larvae were injected with the transfection solution, pcDNA3.0-pre-miR-2845, the mimic, and the inhibitor, after being incubated in the transfection solution. Larvae injected with pcDNA3.0-*egfp*, mimic NC, and inhibitor NC served as controls, and we used three larvae and three replicates for each treatment. The larvae were fed with fresh mulberry as normal, and the PSGs of each group were collected. Total RNAs were extracted and reverse transcribed into cDNAs at 48 h post injection. Specific primers were designed (Table 1) to amplify *BmFib-L*. *BmFib-L* expression levels were detected with qPCR using the cDNAs as templates for analyzing the regulatory effects of bmo-miR-2845 on *BmFib-L*.

### 2.9 Statistics analysis

Gene sequence alignment was performed with DNAMAN software. Spss17.0 software was used to analyze significant differences between treatments (groups). The data of this experiment comes from 3 biological experiments with 3 replicates each time. The *, **, and *** represent significant differences at the *P* < 0.05, *P* < 0.01 and *P* < 0.001 level, respectively.

## 3. Results

### 3.1 There is a target site of miR-2845 on *BmFib-L* 3’UTR

**Fig 1:**
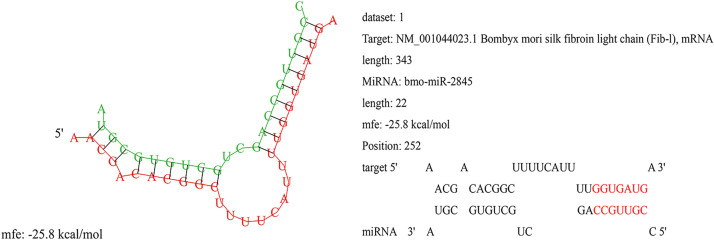
Prediction of bmo-miR-2845 as a potential binding site at 3’UTR of *BmFib-L* mRNA.

The RNAhybrid software predicted bmo-miR-2845 would have potential binding sites at the 3’ UTR of *BmFib-L*. Its minimum free energy was—25.8kcal/mol, with completely complementary pairing of 2–8 bases within the seed sequence region (Fig 1). Thus, miR-2845 was the subject in the following experiments.

### 3.2 Analysis of expression profile of bmo-miR-2845 and *BmFib-L*

**Fig 2:**
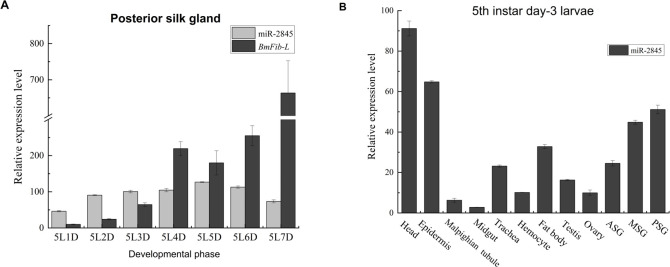
Expression analysis of bmo-miR-2845 and *BmFib-L* in 5th instar larvae of *B*. *mori*. A. Expression levels of miR-2845 and *BmFib-L* in the posterior silk gland of different phases of *B*. *mori*. B. Expression levels of bmo-miR-2845 in different tissues of the 5th instar day-3 *B*. *mori* larvae. Note: Data are represented as the mean ± SD from three independent experiments. The same in the figures below.

bmo-miR-2845 was cloned using specifically designed primers, which were consistent with those listed in the NCBI. Designed primers were also used in subsequent experiments. RT-qPCR showed that bmo-miR-2845 expression increased from the 1st to 5th day in the 5th instar (with a peak on day-5) and decreased thereafter; while the expression of *BmFib-L* increased sharply. This finding indicates that miR-2845 and *BmFib-L* expression levels show opposing trends, which implies a negative regulatory relationship (Fig 2A). miR-2845 was expressed in various tissues on day-3 of the larvae, particularly the head, epidermis and PSGs (Fig 2B). This result suggests that that bmo-miR-2845 is an important miRNA that not only regulates *BmFib-L* in PSG, but also regulate other target genes.

### 3.3 Construction of pre-miR-2845 and *BmFib-L* 3’UTR expression vectors

**Fig 3:**
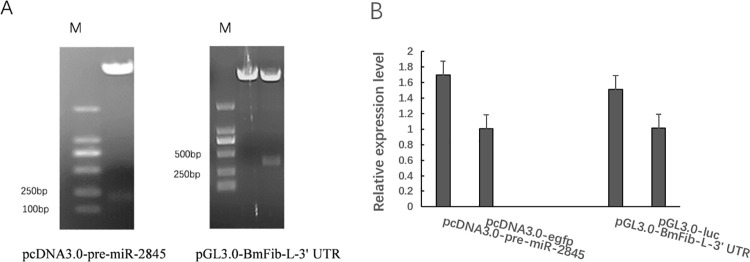
Construction of pre-miR-2845 and *BmFib-L* 3’UTR expression vectors. A. Dentification of pcDNA3.0-pre-miR-2845 and pGL3.0-*BmFib-L-*3’UTR by double enzyme digestion. B. Overexpression verification of pre-miR-2845 and *BmFib-L* 3’UTR.

pre-miR-2845 and *BmFib-L* 3’UTR sequences were amplified with specific primers and cloned into PMD-19T vectors. The correct sequence was cloned into the expression vectors (namely, pcDNA3.0-pre-miR-2845 and pGL3.0-BmFib-L). Through double enzyme digestion and electrophoresis, it was identified that the target fragment was successfully inserted into the vector (Fig 3A). The vectors pcDNA3.0-pre-miR-2845 and pGL3.0-BmFib-L were transfected respectively, and the results showed that the expression levels of miRNA-2845h and BmFib-L both increased by qPCR. Experiments show that the recombinant vector is successfully constructed (Fig 3B).

### 3.4 Bmo-miR-2845 downregulates expression of *BmFib-L* in BmN cells

**Fig 4:**
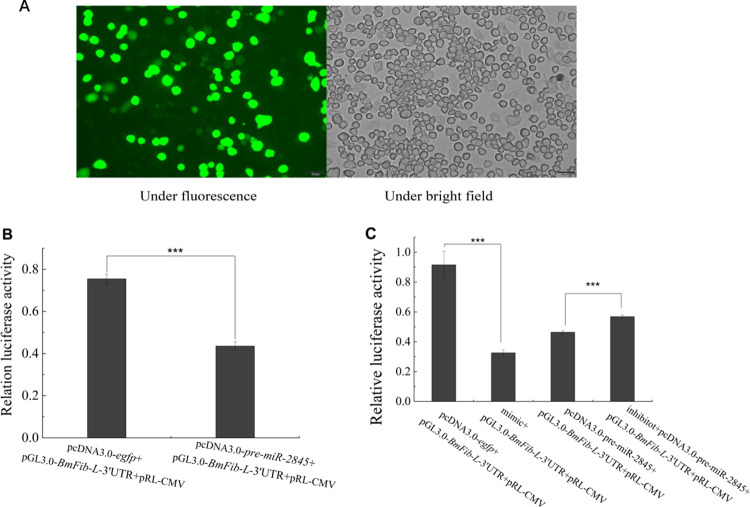
Regulatory function of bmo-miR-2845 on expression levels of *BmFib-L* in BmN cells. A. Green fluorescence in BmN cells (observed at 48 h post-transfection with recombinant plasmids). B. Comparison of luciferase activity in BmN cells of treatment group, the pcDNA3.0-pre-miR-2845 + pGL3.0-*BmFib-L-*3’UTR + pRL-CMV group; the control group, and the pcDNA3.0-*egfp* + pGL3.0 -*BmFib-L-*3’UTR + pRL-CMV group. C. Comparison of luciferase activity in BmN cells of different groups, including the mimic + pGL3.0-*BmFib-L-*3’UTR + pRL-CMV group; the inhibitor + pcDNA3.0-pre-miR-2845 + pGL3.0-*BmFib-L-*3’UTR + pRL-CMV group; the pcDNA3.0-*egfp* + pGL3.0-*BmFib-L*-3’UTR + pRL-CMV group; and the pcDNA3.0-pre-miR-2845 + pGL3.0-*BmFib-L*-3’UTR + pRL-CMV.

BmN cells were co-transfected within the treatment and control groups, and transfection efficiency was observed after 48 h. About 30% of the total cells showed green fluorescence (Fig 4A), indicating that the recombinant vectors were successfully transfected and expressed in the BmN cells. Dual luciferase activities were detected in different groups, with the results showing that, compared with the control group, dual luciferase activity in the treatment group was significantly reduced (by about 1.7 times), at extremely significant level (*P* < 0.001, Fig 4B).

Cells from the experimental and control groups were collected separately to detect dual luciferase activity. Results showed that luciferase activity decreased significantly in the mimic group by 2.8 times (*P* < 0.001) compared to the control group, while luciferase activity in the inhibitor group significantly increased by 1.2 times, (*P* < 0.001) compared to the control group (Fig 4C). Results demonstrated that bmo-miR-2845 significantly downregulated the expression of *BmFib-L* in BmN cells, and that the synthetic mimic and inhibitor are biologically active and could be used in subsequent individual experiments.

### 3.5 Mutation of the target site of miR-2845 on the 3’UTR of *BmFib-L*

**Fig 5:**
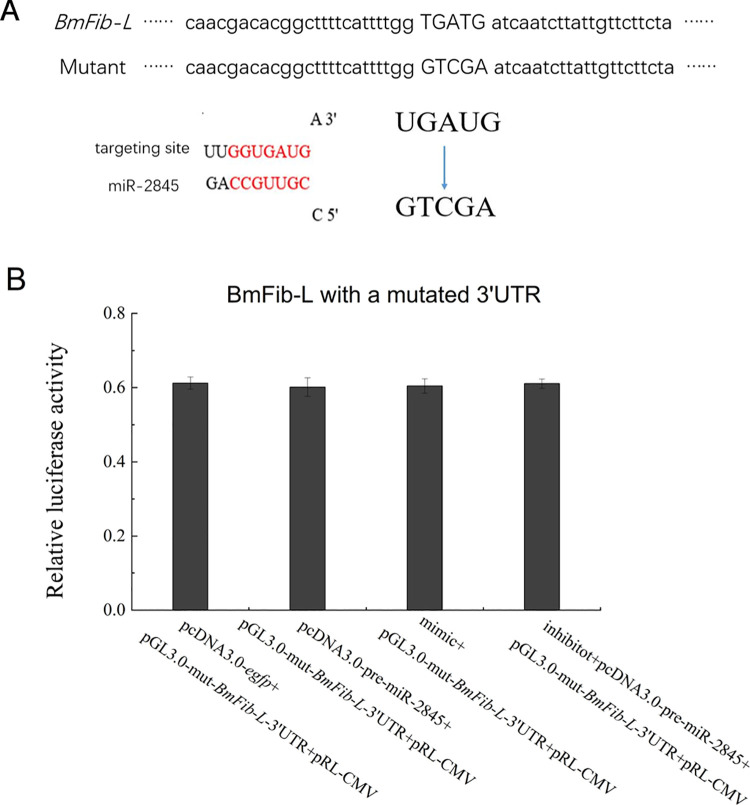
Regulation of bmo-miR-2845 on *BmFib-L* with a mutated 3’UTR. A. Mutant sequence of *BmFib-L*-3’UTR. B. Functional verification of bmo-miR-2845 on *BmFib-L* with a mutated 3’UTR.

The 2nd to 6th bases (sequence: UGAUG) of the predicted miR-2845 targeting site on *BmFib-L* 3’ UTR was mutated to a sequence of GTCGA (Fig 5A). Design four sets of experiments for cell transfection verification. Cells were collected and tested after 48 hours. Luciferase activities showed no significant differences among these four groups (Fig 5B). miR-2845’s inhibitory effects on *BmFib-L* disappeared after mutations, indicating that the predicted target site of bmo-miR-2845 on *BmFib-L* 3’UTR is correct, and that miR-2845 has a regulatory role in the binding site.

### 3.6 Bmo-miR-2845 downregulates expression of *BmFib-L* in *vivo*

**Fig 6:**
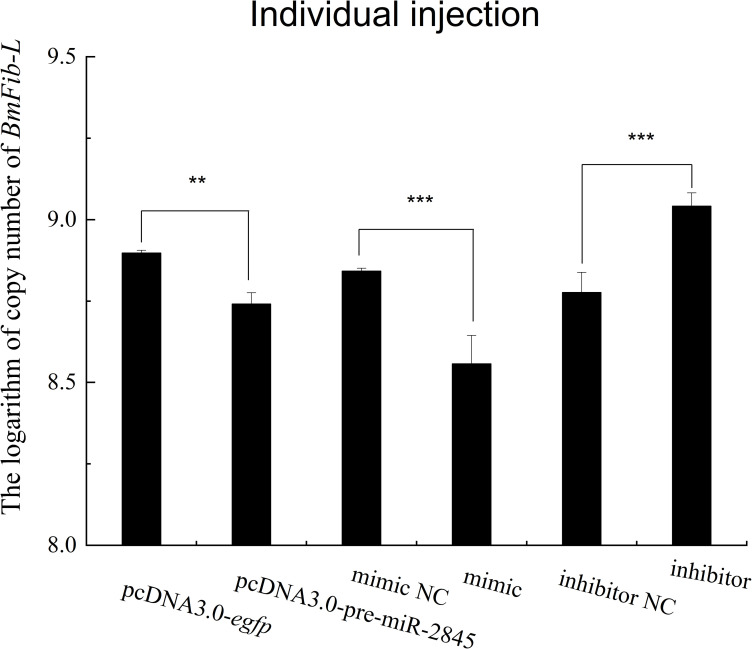
Effects of overexpression and inhibition of bmo-miR-2845 on the expression levels of *BmFib-L* in *B*. *mori* larvae.

Day-2 larvae were injected with the transfection groups mentioned in section 2.8 above. At 48 h post-injection, silk glands were collected, and their total RNAs were extracted for RT-qPCR analysis. The results (Fig 6) showed that overexpression of miR-2845 (pcDNA3.0-pre-miR-2845 transfected larvae) significantly repressed expression of *BmFib-L* (*P*<0.05) compared with those in the pcDNA3.0-egfp transfected group, as did the mimic (*P*<0.001) compared with mimic NC. Further, inhibition of endogenous miR-2845 (inhibitor transfected) significantly increased *BmFib-L* expression (*P*<0.001) compared with that in inhibitor NC. miR-2845’s regulatory function on *BmFib-L* showed the same trend in BmN cells and individuals, adding further evidence that bmo-miR-2845 downregulates *BmFib-L* expression.

## 4. Discussion

There has been an extended focus on *B*. *mori* miRNAs in the literature. More than 600 bmo-miRs have been identified to date. Liu [23] verified 257 bmo-miR genes using Solexa sequencing technology. bmo-miR-2761-3p downregulates the expression of *BmSDH*, which plays an important role in the *B*.*mori* diapause [24]. Song [25] found 29 bmo-miRs related to expression of fibroin genes in the PSG. Functional research around bmo-miRs tends to focus on the regulation of fibroin genes and sericin genes. For example, bmo-miRNA-965 and bmo-miRNA-1926 down-regulate the expression of *BmFib-L* by complementing the target of the 3’UTR [26]. bmo-miR-3377-5p and bmo-miR-2780a separately inhibit *BmSer-1* in expression in cells [27, 28]. While bmo-mir-2805 upregulates *BmFib-L* through *BmAwh* and/or *Bmdimm in vivo* [29]. In this study, we predicted a potential target for bmo-mir-2845 on the *BmFib-L* 3’UTR using RNAhybrid online, confirming that bmo-mir-2845 significantly inhibited the expression of *BmFib-L* at the cellular and individual levels, which has perfected the regulatory network of expression of *B*. *mori* silk protein genes.

*BmFib-L* has strict tissue and developmental specificity. It is mainly expressed in PSGs and the growth stage, but is inhibited in molting stage [30]. As we know, *B*. *mori* larvae produce huge amounts of silk proteins. For example, silk fibroin genes and sericin genes are highly expressed, especially in the 5th instar [31]. Previous studies have shown that expression of *BmFib-L* in the posterior silk glands increases continuously during the development of the 5th instar [32]. Our experimental results also show that *BmFib-L* is highly expressed in the later stages of 5th instar compared to the early and middle stages.

Since a miRNA can target to multiple genes, miRNA clusters can influence many cellular functions by regulating different targets. For example, the mir-17-92 cluster is not only involved in tumor formation, but also in the normal development of the heart, lung and immune system [33–35]. In our experiment, the high expression of miR-2845 is in the head, indicating that miR-2845 can target genes in the head outside of *BmFib-L* in the PSG. Studies have shown that the head of insects is nerve control center. Further, the hormone peptides produced by the nerve secreting cells can regulate the insect endocrine and the balance of homeostasis [36]. However, the specific role of miR-2845 in the head remains unclear, and needs to be further studied.

Due to the seed sequence of a miRNA is short, it can match with many sites during gene expression, and may bind with multiple mRNAs to regulate many signaling pathways [37]. In this experiment, the 3’UTR of *BmFib-L* was set as the target sequence, and was verified by target mutation experiments. However, it remains unknown whether bmo-miR-2845 can bind to other genes, and, if it does, what function it may be serving. Thus, further study on bmo-miR-2845 is still needed.
